# Prevalence of SARS-CoV-2 Infection among Children and Adults in 15 US Communities, 2021^1^

**DOI:** 10.3201/eid3002.230863

**Published:** 2024-02

**Authors:** Jessica Justman, Timothy Skalland, Ayana Moore, Christopher I. Amos, Mark A. Marzinke, Sahar Z. Zangeneh, Colleen F. Kelley, Rebecca Singer, Stockton Mayer, Yael Hirsch-Moverman, Susanne Doblecki-Lewis, David Metzger, Elizabeth Barranco, Ken Ho, Ernesto T.A. Marques, Margaret Powers-Fletcher, Patricia J. Kissinger, Jason E. Farley, Carrie Knowlton, Magdalena E. Sobieszczyk, Shobha Swaminathan, Domonique Reed, Jean De Dieu Tapsoba, Lynda Emel, Ian Bell, Krista Yuhas, Leah Schrumpf, Laura Mkumba, Jontraye Davis, Jonathan Lucas, Estelle Piwowar-Manning, Shahnaz Ahmed, the CoVPN 5002 COMPASS Study Team

**Affiliations:** Columbia University, New York, New York, USA (J. Justman, Y. Hirsch-Moverman, M.E. Sobieszczyk, D. Reed);; Fred Hutchinson Cancer Center, Seattle, Washington, USA (T. Skalland, S.Z. Zangeneh, J. De Dieu Tapsoba, L. Emel, I. Bell, K. Yuhas);; FHI 360, Durham, North Carolina, USA (A. Moore, L. Schrumpf, L. Mkumba, J. Davis, J. Lucas);; Baylor College of Medicine, Houston, Texas, USA (C.I. Amos);; Johns Hopkins University, Baltimore, Maryland, USA (M.A. Marzinke, J.E. Farley, E. Piwowar-Manning, S. Ahmed);; Emory University, Atlanta, Georgia, USA (C.F. Kelley);; University of Illinois Chicago, Chicago, Illinois, USA (R. Singer, S. Mayer);; University of Miami, Miami, Florida, USA (S. Doblecki-Lewis);; University of Pennsylvania, Philadelphia, Pennsylvania, USA (D. Metzger);; Ponce Health Sciences University, Ponce, Puerto Rico (E. Barranco);; University of Pittsburgh, Pittsburgh, Pennsylvania, USA (K. Ho, E.T.A. Marques);; University of Cincinnati, Cincinnati, Ohio, USA (M. Powers-Fletcher);; Tulane University, New Orleans, Louisiana, USA (P.J. Kissinger);; University of Colorado, Aurora, Colorado, USA (C. Knowlton);; Rutgers Medical School, Newark, New Jersey, USA (S. Swaminathan);; RTI International, Research Triangle Park, North Carolina, USA (S.Z. Zangeneh)

**Keywords:** COVID-19, 2019 novel coronavirus disease, coronavirus disease, severe acute respiratory syndrome coronavirus 2, SARS-CoV-2, viruses, respiratory infections, zoonoses, time/location sampling, community surveys, surveys and questionnaires, epidemiology, United States

## Abstract

During January–August 2021, the Community Prevalence of SARS-CoV-2 Study used time/location sampling to recruit a cross-sectional, population-based cohort to estimate SARS-CoV-2 seroprevalence and nasal swab sample PCR positivity across 15 US communities. Survey-weighted estimates of SARS-CoV-2 infection and vaccine willingness among participants at each site were compared within demographic groups by using linear regression models with inverse variance weighting. Among 22,284 persons >2 months of age and older, median prevalence of infection (prior, active, or both) was 12.9% across sites and similar across age groups. Within each site, average prevalence of infection was 3 percentage points higher for Black than White persons and average vaccine willingness was 10 percentage points lower for Black than White persons and 7 percentage points lower for Black persons than for persons in other racial groups. The higher prevalence of SARS-CoV-2 infection among groups with lower vaccine willingness highlights the disparate effect of COVID-19 and its complications.

## Abstract 2

Higher infection prevalence and lower vaccine willingness underscore the need for more effective prevention and vaccine strategies in communities at risk.

As of May 2023, ≈104 million confirmed SARS-CoV-2 cases had been reported in the United States (*1*). That case count is certainly an underestimate, given the occurrence of asymptomatic disease; self-testing and unreported cases; and limited initial diagnostic testing, especially among children. The true case count may be gleaned from SARS-CoV-2 seroprevalence studies. In most parts of the world, including the United States, many prevalence estimates have been based on convenience samples of adults (*2*), including samples from healthcare settings (*3*) or from US commercial laboratories (*4*). According to those approaches, seroprevalence has varied from 10% to 58%, depending on the type of serologic test used, calendar time in relation to the pandemic, population sampling strategy, and characteristics of the population (e.g., demographic, clinical, and healthcare seeking) (*3*,*4*).

Population-based seroprevalence estimates from nonclinical general populations have been few (*5*), reflecting challenges posed by the COVID-19 pandemic with regard to rigorous sampling strategies for reaching representative populations (*6*–*9*). Some strategies have used social media to recruit diverse populations but lacked a well-defined sampling frame (*10*), and regional studies with random sampling schemes have lacked diverse participation (*11*–*13*). In addition, many seroprevalence studies have not included detailed demographic and socioeconomic information about the participants despite the association of those factors with SARS-CoV-2 infection (*14*–*17*).

We report the results of the Community Prevalence of SARS-CoV-2 Study (COMPASS), which was conducted in the first half of 2021 to assess prevalence of prior and current SARS-CoV-2 infection among the general population of adults and children in largely urban communities surrounding established clinical research sites in the United States. We based determination of infection on antibody and PCR positivity. We also describe the population-level factors associated with increased risk for SARS-CoV-2 infection. COMPASS used time/location sampling (TLS) as a rigorous method of nonprobability sampling of public venues near the participating research sites to enroll persons from the community (*18*,*19*). To improve the representativeness of the general population sample given the mobility restrictions of the pandemic, COMPASS also recruited a clinical cohort from outpatient healthcare facilities and a nursing home cohort from residential facilities for older adults.

## Methods

### Study Design and Setting

COMPASS (ClincialTrials.gov identifier NCT04658121, https://clinicaltrials.gov) was a cross-sectional survey sponsored by the National Institute of Allergy and Infectious Diseases (NIAID)–funded COVID-19 Prevention Network (*20*). A total of 68 existing US-based NIAID clinical research sites (hereafter called sites) were invited to conduct the study with the goal of enhancing representative sampling in multiple regions across the United States. The 15 participating sites (Appendix) were located in the southern, midwestern, mid-Atlantic, and northeastern United States as well as Puerto Rico.

Ethics approvals were obtained from a central (Advarra) and participating sites’ institutional review boards (Appendix). All participants or their representatives provided written informed consent or assent for persons 7–17 years of age with parental/guardian consent; remote electronic consent for persons 15–17 years of age was permissible for parents not physically present. For adults with mental incapacity, consent was provided by a legally authorized representative.

### Participants and Sampling

The study population consisted of adults and children recruited from the catchment area of each site (*18*). TLS was used to recruit participants in the community and outpatient clinic cohorts, and because of pandemic restrictions to access, convenience sampling was used to recruit the nursing home cohort. Ethnographic mapping of community venues in each catchment area (e.g., supermarkets, parks, commercial streets, and interviews with venue managers when relevant) identified time venues (i.e., times when venues were available and likely to have foot traffic). Clinical venues where persons visiting outpatient facilities were recruited to the clinical cohort were distinct from community venues. The catchment area of each site was defined as the postal (ZIP) code of the site plus all contiguous ZIP codes, encompassing a population of ≈150,000 (Appendix).

Research teams visited time venues randomly selected on the basis of sampling frames updated weekly by each site and attempted to recruit all persons at each time venue (Appendix). They collected the number of persons approached versus enrolled at each time venue, and the resulting ratio was used to adjust for nonresponse. Adults >18 years of age and children >2 months of age who were recruited in the community were eligible for the community cohort; adults who were recruited at a selected outpatient healthcare facility were eligible to join the clinical cohort; and those recruited at a senior living facility were eligible to join the nursing home cohort. For all cohorts (community, outpatient, senior living facility), potential participants were excluded if they had previously enrolled in the study or if there was any condition that, in the opinion of the study staff, would interfere with achieving the study objectives. COVID-19 vaccination was not exclusionary. Study participants received a cash gift card consistent with local standards as compensation for time and effort.

We collected demographic, socioeconomic, geographic, clinical, and household SARS-CoV-2 exposure and infection history information from each consenting participant (or a parent of participants <9 years of age) via an interviewer-administered questionnaire conducted in English or Spanish in a relatively private location at the venue (e.g., to the side of the tent). The questionnaire assessed COVID-19 symptoms and willingness to receive an approved COVID-19 vaccine. At the end of March 2021, the questionnaire was updated to allow participants to self-report whether they had received an approved vaccine (i.e., a US Food and Drug Administration–authorized COVID-19 vaccine).

Participants provided whole blood samples via venipuncture and mid-turbinate samples via nasal swabbing. For participants <2 years of age, blood was collected by heel or finger stick, and dried blood spots were prepared at study sites. Serum was isolated from blood, and serologic evidence of prior SARS-CoV-2 infection was evaluated by using the Abbott Architect SARS-CoV-2 IgG nucleocapsid antibody assay (Abbott Diagnostics), for which, according to manufacturer claims, specificity was 99.6% and sensitivity was 100% (*21*). To reduce interassay and interlaboratory variability, testing was performed at a central laboratory (Quest Diagnostics) by use of a single assay. To determine the prevalence of active SARS-CoV-2 infections, PCR analysis was performed on midturbinate nasal swab samples by using validated, approved assays (Appendix). SARS-CoV-2 PCR results, but not serologic results, were returned to study participants.

### Outcomes

The primary outcome was the proportion of participants with prior SARS-CoV-2 infection, based on presence of SARS CoV-2 IgG nucleocapsid antibody (Ab+). A secondary outcome was the proportion of participants with active SARS-CoV-2 infection, based on results of SARS-CoV-2 RNA testing (PCR+). A combined outcome of the proportion with prior or active SARS-CoV-2 infection was based on having a status of Ab+, PCR+, or both. Vaccine willingness was defined as the proportion of participants who reported on a 5-point scale that they were likely or very likely to receive an approved vaccine or responded that they had already received an approved vaccine. The proportion of Ab+ participants who reported being asymptomatic was based on the number of participants who responded no to all 13 yes/no questions about experiencing upper respiratory or systemic symptoms since November 2019. The proportion of PCR+ participants who reported being asymptomatic was similarly based on the number who responded no when asked about each of the same 13 symptoms currently or in the past 14 days (Appendix).

### Power Calculation and Statistical Analyses

The target sample size for each of the 4 target age groups (<18, 18–39, 40–59, >60 years) in the community cohort was 730, based on a prespecified margin of error of 2.5% for seroprevalence of <5% and a 5% margin of error for seroprevalence of 10%–25% (*18*). Each site therefore sought to enroll 2,920 participants from community venues, as well as an additional 500 adults from outpatient health facilities and 500 adults from nursing homes.

We constructed estimates for the 3 laboratory-based prevalence outcomes as well as vaccine willingness for each of the 15 clinical research site communities overall; for each target age group; and by sex assigned at birth (female, male), race (Black, White, other), and ethnicity (Hispanic, non-Hispanic). Other race categories were Asian, Native Hawaiian or other Pacific Islander, and other, as well as those answering don’t know/not sure and prefer not to answer. Prevalence by gender identity was not estimated because 68% of enrolled participants did not respond to a question about current gender identity. Survey weights accounted for sampling design (*18*), nonresponse, and, per data from the American Community Survey (https://data.census.gov/table/?d=ACS%205-Year%20Estimates%20Detailed%20Tables), poststratification (Appendix).

We limited analyses of cross-site summary measures of the combined endpoint (Ab+, PCR+, or both) and vaccine willingness to sites that had enrolled >25 participants in the specific demographic group and described those measures as medians with interquartile ranges (IQRs). We separately compared combined prevalence outcome (active infection, prior infection, or both) and vaccine willingness within demographic groups (age, sex, race, and ethnicity) by using linear regression models with inverse variance weighting. We used a robust heteroskedasticity-consistent type sandwich variance estimation approach to account for potential nonconstant error variances in the weighted linear regression models that included the proportion (e.g., vaccine willingness) as an outcome and the demographic variable of interest as a covariate while accounting for site.

## Results

**Figure 1:**
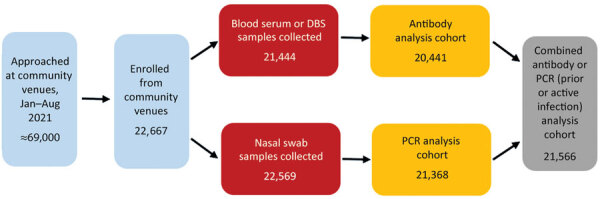
Participant enrollment from community venues in study of prevalence of SARS-CoV-2 infection among children and adults in 15 US communities (COMPASS 2021). Each site completed community enrollment from a median of 30 (interquartile range 24–35) venues. At each site, 80% of community enrollments were completed at a median of 13 (interquartile range 8.5–15.5) venues. DBS, dried blood spot.

**Figure 2:**
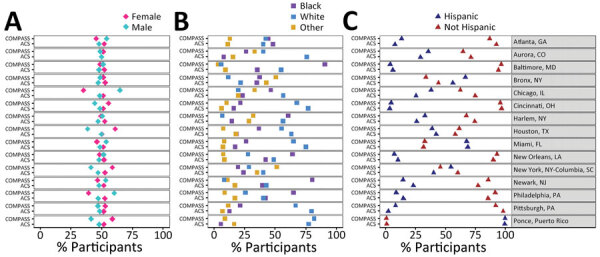
Demographic characteristics of community cohort (n = 21,189) in study of prevalence of SARS-CoV-2 infection among children and adults in 15 US communities (COMPASS 2021) and the 2020 US Census American Community Survey (https://www.census.gov/programs-surveys/acs) for each site.

We enrolled 26,201 adults and children in the study from January 12, 2021, through August 12, 2021; median recruitment period per site was 164 days (range 84–199 days, IQR 150–185 days). During that time, ≈69,000 persons were approached from a cumulative total of >450 unique community venues surrounding the 15 clinical research sites; 22,284 (≈32%) participants enrolled (Figure 1), resulting in a median enrollment per community of 1,246 (range 508–2,924, IQR 997–1,682) participants. Sites intended to enroll one quarter of the community cohort into each of the 4 age groups; however, only 2,113 (9.5%) enrolled participants were <18 years, and the median number of children per site was 48 (IQR 24–100). Most sites enrolled similar proportions of male and female participants from community venues (Appendix Table 2). A total of 3,111 participants were enrolled in the clinical cohort and 806 in the nursing home cohort (Appendix Tables 1, 3, 4, Figures 1, 2).

A total of 22,284 participants of the targeted 43,800 were enrolled from community venues; of those, 95% provided a blood sample for SARS-CoV-2 antibody testing and 99% provided a nasal swab sample for SARS-CoV-2 PCR testing (Figure 1). Complete data were available for 21,189 (95%) community cohort enrollees for analysis of a combined endpoint of Ab+, PCR+, or both (i.e., evidence of prior or active infection). The remainder of the findings about the community cohort pertain to the group of 21,189.

The unadjusted demographic profile for sex and ethnicity of the community cohort was similar to the estimated demographic profiles for most communities according to corresponding county-level data from the American Community Survey (Figure 2); however, the profile by race differed at several sites. In addition, prevalence of race varied widely, from 9% to 89% for Black persons (median 37% [IQR 24%–62%] across sites) and from 6% to 80% for White persons (median 28% [IQR 15%–60%] across sites). Hispanic ethnicity varied from 3% to 98% (median 33% [IQR 9%–47%] across sites).

**Figure 3:**
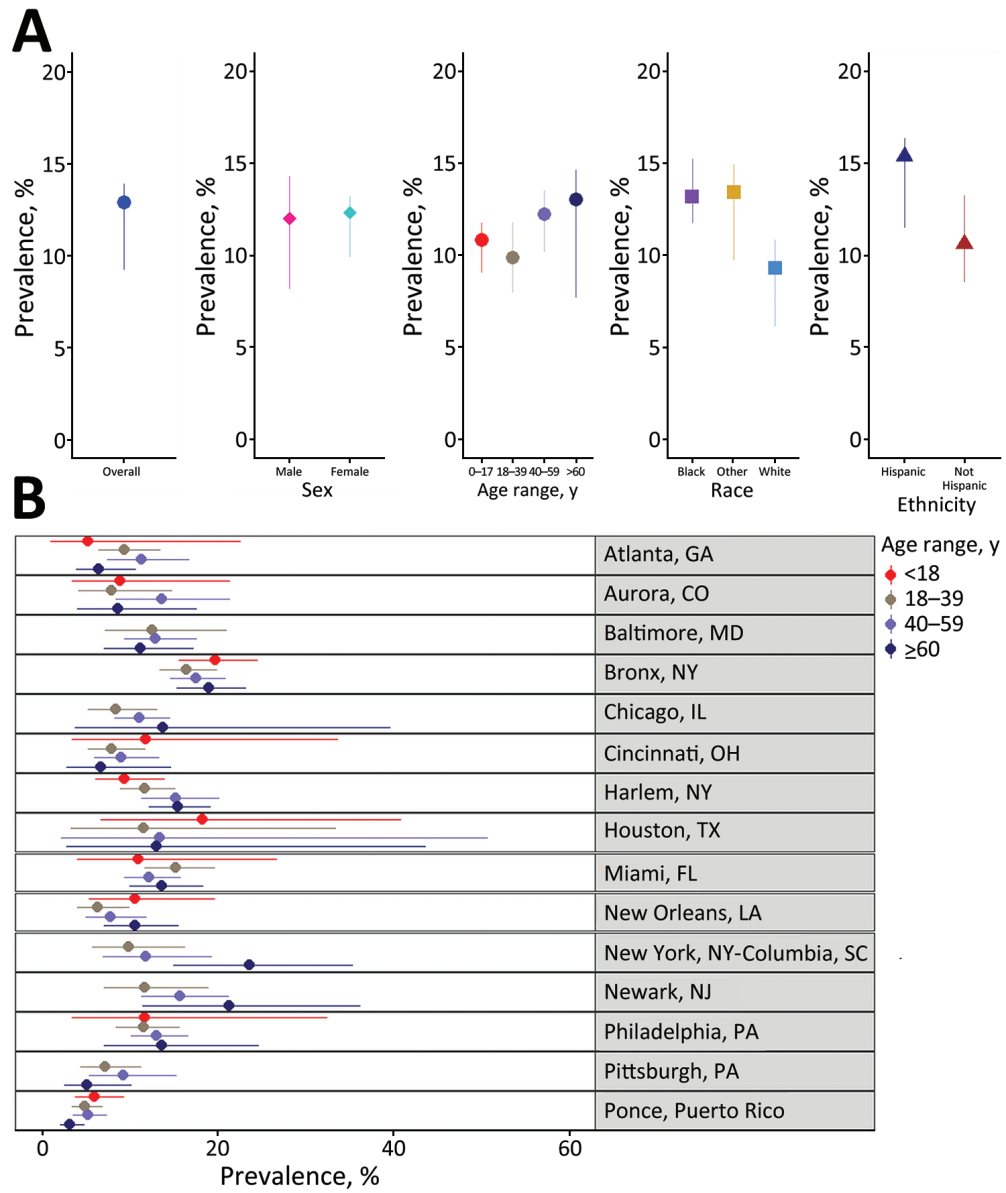
Prevalence of active or prior COVID-19 infection in 15 US communities, community cohort (n = 21,189), COMPASS 2021. A) By demographic characteristic across all sites. B) By age and site. Error bars indicate interquartile ranges. Note: Analysis of age limited to communities with >25 children who had complete data.

Median seropositivity of the community cohort (Appendix Table 5, Figure 3), determined according to 2,272 Ab+ participants, was 12.4% (IQR 9.1%–14%); seropositivity was similar for the clinical cohort (11.3% [IQR 7.7%–15.5%]) but lower for the nursing home cohort (3.3% [IQR 2.4%–7.7%]). Of note, 50% of the nursing home cohort participants were recruited in Puerto Rico, where seroprevalence for all age groups was 3%–5% (Figure 3, panel B). The median overall prevalence of active infection (PCR positivity) in the community cohort across all sites (Appendix Table 5, Figure 3), determined according to 189 PCR+ participants, was 0.8% (IQR 0.2%–1.5%) A total of 64 participants enrolled in COMPASS were both PCR+ and Ab+ (52 from the community cohort and 12 from the clinical cohort).

In the community cohort, overall median prevalence across all 15 site-level estimates of the combined endpoint, based on PCR+ or Ab+ status, was 12.9% (range 4.9%–18%, IQR 9.2%–14%) (Figure 3, panel A). Although prevalence varied widely, when we looked at demographic differences within each site, we found no difference in average prevalence of prior or active infection by age, whether considering the 4 age groups at all sites (Figure 3, panel A) or comparing children (<18 years of age) with adults (>18 years of age) at each of the 10 sites that enrolled >25 children (Figure 3, panel B). We also found no difference in average prevalence by participant sex or ethnicity (Figure 3, panel A). Within each site, however, the average prevalence estimate for Black participants was 3 percentage points higher than for White participants (p<0.01) and 2.4 percentage points higher than for those with race identified as other (p = 0.11). Among participants >18 years of age, we found a nonsignificant association between a higher prevalence of active or prior SARS-CoV-2 infection and a lower level of attained education (Appendix Figure 4). We found no association between COVID-19 and income level; however, many (32.8%) participants reported their household income as don’t know or prefer not to answer (data not shown).

About half of seropositive persons in the community cohort reported that they had not ever experienced COVID-19 symptoms (median 50.0% [IQR 45.3%–63.8%]; data not shown). Similarly, about half of PCR+ persons in the community cohort reported not having experienced symptoms within the 14 days before their participation in the survey (median 52.4% [IQR 50.0%–62.4%]; data not shown). In contrast, among the seronegative persons in the community cohort, a median of 80.9% (IQR 75.7%–83.4%; data not shown) reported they had not ever experienced COVID-19 symptoms. Similarly, a median of 79.8% of PCR-negative persons (IQR 75.7– 83.1%; data not shown) in the community cohort reported not having experienced symptoms within the 14 days before the survey.

In a separate analysis of self-reported PCR and antibody results among the 21,940 persons who enrolled and tested antibody negative in COMPASS (18,629 from the community cohort), prevalence of a self-reported prior positive COVID-19 test (nasal swab sample) was 4.9% (n = 1,067) and prevalence of a self-reported prior positive antibody test was 2.4% (n = 526) (data not shown). By contrast, among the 2,792 who enrolled and tested antibody positive in COMPASS (2,380 from the community cohort), prevalence of a self-reported prior positive COVID-19 test (nasal swab sample) was 33.4% (n = 933) and prevalence of a self-reported prior positive antibody test was 7.9% (n = 221) (data not shown).

**Figure 4:**
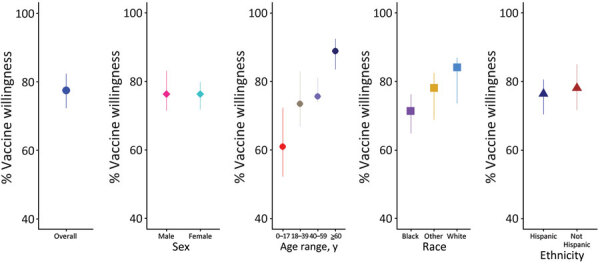
Prevalence of COVID-19 vaccine willingness in 15 US communities, by demographic characteristics, all sites, community cohort, COMPASS 2021. Note: Analysis of age limited to communities with >25 children who had complete data.

Overall, across 15 site-level estimates, the median percentage of persons in the community cohort who reported being willing to receive a COVID-19 vaccine was 78% (IQR 72%–82%); the percentage was higher among participants >60 years of age (median 89%, IQR 84%–92%) compared with those 18–39 years of age (median 74%, IQR 67%–83%) and 40–59 years of age (median 76%, IQR 74%–81%) (Figure 4). The median percentage of participants who reported being willing to receive a vaccine was 71.4% (IQR 64.8%–76.3%) for Black, 78% (IQR 68.8%–82.5%) for other race, and 84.2% (IQR 73.6%–87.0%) for White participants (Appendix Table 6). We found no differences in willingness to receive a vaccine by sex or ethnicity across the sites. When looking at demographic differences within each site, we found that vaccine willingness among those >60 years of age was an average of 10 percentage points higher than among those 40–59 (p<0.01) years of age, 11 percentage points higher than those 18–39 (p<0.01) years of age, and 22 percentage points higher than those <18 years of age (p<0.01). Similarly, the average difference among participants who reported being willing to receive a vaccine was 10 percentage points lower for Black compared with White participants (p<0.01) and 7 percentage points lower for Black participants than for persons in other racial groups (p<0.01).

## Discussion

COMPASS, a population-based cross-sectional serosurvey, enrolled ≈22,000 adults and children from community venues in 15 largely urban US settings in the first half of 2021 and found that the overall prevalence of prior and active SARS-CoV-2 infection was 12.9%. TLS, typically used to recruit hard-to-reach populations (*19*), was an innovative way to randomly sample populations representative of diverse communities amid pandemic restrictions to access and movement, based on demographic comparisons with the American Community Survey. Contrasting with initial reports of COVID-19 (*22*–*24*), population-based prevalence did not vary by age, indicating that acquisition was similar for all age groups. In addition, despite prevalence of prior or active COVID-19 being higher among Black participants, fewer Black participants in the sampled communities were willing to receive an approved COVID-19 vaccine compared with participants who were from White or other racial groups, a finding that may have magnified the disparate burden of COVID-19 and associated outcomes in these communities.

Compared with our findings, studies conducted in healthcare settings may have arrived at higher estimates of COVID-19 prevalence (*3*,*4*), reflecting the symptomatic status, healthcare access, and healthcare-seeking behavior of clinical populations. For example, a commercial laboratory seroprevalence study conducted in the United States during the second half of 2021 assessed at 4-week intervals convenience samples of blood specimens collected for clinical testing and found an overall US seroprevalence of 33.5% in December 2021 (*4*). Similar to our findings with regard to age, the study of commercial laboratory samples also found that seroprevalence among children and adults did not differ. Whether for COVID-19 or other widespread outbreaks, such as mpox, seroprevalence estimates from population-based studies that include those who are less likely to engage in care, combined with estimates from healthcare settings, may offer the most comprehensive picture of outbreak effects at that point in time.

COMPASS found that willingness to receive a vaccine during the first half of 2021, during the earliest stages of vaccine availability in the United States, was 10 percentage points lower among Black participants and 7 percentage points lower among other race participants than among White participants. Monthly telephone surveys of immunization practices, conducted during December 2020–November 2021, found a similar level of racial disparity in April 2021 (*25*). By November 2021, racial disparities in COVID-19 vaccine uptake in the United States were no longer evident (*26*); nonetheless, 1 year later, by December 2022, Black race and Hispanic ethnicity remained associated with higher rate ratios of age-adjusted hospitalization and death compared with White race (*26*,*27*), indicating that vaccine uptake alone did not explain disparities in outcomes.

Strengths of our study included collection of biological samples and detailed demographic, behavioral, and self-reported clinical data from randomly sampled persons from the general population, including children. COMPASS used a centrally managed approach to TLS to ensure a participant population that reflected the communities surrounding clinical research sites participating in COVID-19 vaccine trials. The weekly systematic updating of community venue sampling frames was a novel way to adapt to changing COVID-19 guidelines and restrictions as well as to fluctuating weather (*18*). The prevalence of active infection was based on validated laboratory assays performed on approved platforms. Although we did not assess serologic evidence of vaccine immunity (SARS-CoV-2 spike antibody), the serologic assessment of prior infection through a single assay conducted at a centralized laboratory yielded rigorous seroprevalence findings.

Limitations of our study included conducting it in the first half of 2021, just before the Delta variant became dominant in the United States, when vaccines were becoming available, home-based rapid test kits were not yet widely available, and even PCR testing was often not easily available. Although enrollment targets, especially for children, were not fully achieved, the study had sufficient power to compare both seroprevalence and the combined endpoint of seroprevalence, PCR positivity, or both, by age group. The results do not reflect the entire US population but do reflect persons who were well enough to attend commonly frequented venues in diverse, largely urban communities surrounding the participating clinical research sites, who represent those who had more exposure to COVID-19 compared with those who were homebound. Sampling largely urban settings was relevant, given that the US population is 80% urban (*28*) and that, globally, COVID-19 cases had a large effect in urban settings, especially in the earlier stages of the epidemic (*29*). Serologic testing may have underestimated seroprevalence because of either absent or low titers after a mild infection (*30*) or waning antibody titers after a distant (>6 months) infection (*31*). Last, recall and social desirability biases may have affected the accuracy of some replies to some sections of the questionnaire.

In our population-based survey, we used TLS to recruit members of the general population who were relatively hard to reach because of the status of the COVID-19 pandemic. Our findings demonstrate that prior and active SARS-CoV-2 infection varied widely by community but, contrasting with initial reports, not by age. Half of Ab+ and PCR+ participants reported no symptoms, underscoring the limitations of case-based reporting and the potential for asymptomatic transmission (*32*,*33*). Our findings of higher prevalence of prior or active COVID-19 among persons who were less willing to get vaccinated highlight the value of tailoring public health efforts to the communities most likely to continue to experience disparate effects COVID-19 and its complications.

Appendix Additional information for study of prevalence of SARS-CoV-2 infection among children and adults in 15 US communities.
