# Supplementary material for: Prevalence of SARS-CoV-2 Infection among Children and Adults in 15 US Communities, 2021
Source: Emerg Infect Dis. 2024 Feb;30(2):245–54. doi: 10.3201/eid3002.230863 (PMC10826749; doi:10.3201/eid3002.230863)
Supplement: Appendix — Additional information for study of prevalence of SARS-CoV-2 infection among children and adults in 15 US communities. [file 23-0863-Techapp-s1.pdf]

Article DOI: https://doi.org/10.3201/eid3002.230863

EID cannot ensure accessibility for supplementary materials supplied by authors. Readers who have difficulty accessing supplementary content should contact the authors for assistance.

Prevalence of SARS-CoV-2 Infection among Children and Adults in 15 US Communities

Appendix

Research sites: The Ponce de Leon Center Clinical Research Site (CRS), Atlanta, GA; Children's Hospital Colorado CRS, Aurora, CO; Johns Hopkins CRS, Baltimore, MD; Bronx Prevention Research Center CRS, NY, NY; UIC Project Wish CRS, Chicago, IL; Cincinnati CRS, Cincinnati, OH; Harlem Prevention Center CRS, NY, NY; Baylor College of Medicine, Houston, TX; University of Miami Infectious Disease Research Unit at Jackson Memorial Hospital CRS, Miami, FL); New Orleans Adolescent Trials Unit CRS, New Orleans, LA; Columbia P&S CRS, NY, NY; New Jersey Medical School CRS, Newark, NJ; Penn Prevention CRS, Philadelphia, PA; University of Pittsburgh CRS, Pittsburgh, PA; St. Louis University VTEU, CAIMED-PHSU, Ponce, Puerto Rico.

Recruitment: Information on the venue-day-times (VDTs) attempted and attended were documented and uploaded as end-of-day information. Field teams were instructed to use their judgement when recruiting from venues and to include all individuals present. Depending on the size of the crowd, staff greeted and attempted to engage with all individuals who came into the recruitment space and inquired about the individual’s interest in learning about and enrolling in the study. If the numbers of individuals were too high to do this in a meaningful way, staff were instructed to decide at the beginning of and throughout the time slot, to greet each nth individual who came into the recruitment space and inquire about their interest in hearing about the study. Staff documented this sampling interval. The number of individuals approached was defined as the number to whom staff were able to deliver part of a standardized prescreening message about COMPASS and included those who declined further engagement and those who agreed to provide informed consent. Staff used a hand counter to track this number and documented at the end of each VDT the number approached and the number enrolled.

Lab methods: To determine the prevalence of active SARS-CoV-2 infections, PCR analysis was performed on mid-turbinate nasal swabs using one of the following platforms, if available: Abbott Molecular/RealTime SARS-CoV-2 assay; Roche/ Cobas®SARS-CoV-2 test; or Hologics Aptima SARS CoV-2 assay. If one of the above assays was not available, the study team reviewed and approved alternative PCR-based platforms. Alternative platforms were used at the following sites: Atlanta (BakoDx Coronavirus (COVID-19) SARS-CoV-2 Test), Baylor (CDC 2019-Novel Coronavirus Real-Time RT-PCR Diagnostic Panel), Penn (Cepheid Xpert® Xpress SARS-CoV-2 test), and Tulane (ThermoFisher Scientific TaqPath™ COVID-19 Combo Kit).

To determine the prevalence of prior SARS-CoV-2 infections, antibody testing was performed at a single laboratory. During the planning phase of the study, in the second half of 2020, there were few laboratories that had the capacity to perform the projected testing of 43,800 samples. Based on laboratory infrastructure, testing was performed at Quest Diagnostics (Horsham, PA) using the Abbott SARS-CoV-2 IgG NC AB assay, a two-step immunoassay that detects IgG antibodies to SARS-CoV-2 via chemiluminescent microparticle immunoassay technology; a chemiluminescent signal to cutoff index greater than or equal to 1.40 was considered positive (*1*). While the assay has demonstrated high positive percent agreement with PCR results as soon as 14 days after onset of symptoms (100%, 95% CI: 95.9; 100.0), antibody signal has also been shown to decrease over time (*2,3*).

Statistical analysis plan: The protocol SAP is provided and includes a description of the construction of the design-based weights for primary and secondary analyses; many of these details have been recently reported (*4*). Domain estimates were used for all of the subgroup analyses. Additional analyses within this paper, not included in the SAP, were as follows:

To estimate the prevalence of a combined active or prior SARS-CoV-2 infection based on results of SARS-CoV-2 RNA testing and IgG seropositivity testing.

Combined active or prior infection was defined as a participant having either SARS-CoV-2 infection or IgG seropositivity (they must have had at least one of the results recorded). Survey weighted estimates were used to obtain the prevalence of individuals with combined active or prior infection. Survey weights were used to reflect the sampling design for each age strata and post-stratified to American Community Survey census data on known demographic

variables. The design-based estimates of the mean and their corresponding standard errors were used to construct 95% confidence intervals.

To assess the association between demographic factors with SARS-CoV-2 infection and seroprevalence in the general population using all research sites.

We first obtained survey weighted estimates and corresponding standard errors for the combined active or prior infection prevalence by demographic group for each site. For each group (e.g., race) we then used those estimates and standard errors in an inverse-variance weighted linear regression that had the demographic variable as a covariate and accounts for research site. Finally, we used a heteroscedasticity-consistent standard error (HC1) to test and estimate differences between groups using *t*-intervals.

To assess vaccine willingness among the general population.

Vaccine willingness was defined as participants responding “Likely” or “Very Likely” to receive an approved vaccine for SARS-CoV-2 or responding that they have already received an approved vaccine. This was assessed as a 5-point Likert scale response (with additional option of “already received”) on the questionnaire administered to enrolled participants. Survey weighted estimates were used to obtain the proportion of individuals willing to receive an approved vaccine for SARS-CoV-2. Survey weights were used to reflect the sampling design for each age strata and post-stratified to census data on known demographic variables. The design-based estimates of the mean and their corresponding standard errors were used to construct 95% confidence intervals.

To assess the proportion of reported asymptomatic participants among those with active or prior infection in the general population.

Among those individuals with active SARS-CoV-2 infection, we used the proportion not reporting symptoms based on Yes/No responses to questions about whether they currently were experiencing or in the past 14 days had experienced any of 13 upper respiratory or systemic symptoms. Participants with missing symptom responses were included in the denominator.

Among those individuals with prior SARS-CoV-2 infection, we used the proportion not reporting symptoms based on a Yes/No response to a question on ever having COVID-19

symptoms since November 2019. Participants with missing or unsure symptom response were included in the denominator.

Survey weighting methodology was not used for either of these estimates.

To assess the proportion of individuals with prior COVID-19 testing among those with prior infection in the general population.

For the analysis of self-reported PCR and Ab testing performed prior to and separately from the COMPASS study, we included all study participants in the denominator, regardless of whether responses to questions on prior PCR and or Ab testing indicated they had never had a test or if the response was missing. Survey weighting methodology was not used for these estimates.

Survey sampling weights:

Sampling design weights were constructed for each site from probabilistic sampling of available days and times for community venues and outpatient facilities, based on the inverse of the inclusion probabilities derived in Section 2.2 of the SAP as base weights.

Non-response was estimated at each venue and outpatient health facility based on the number of people who were approached and the number enrolled by the research team.

Post-stratification adjustments were done only for the community cohort through a raking process (marginal adjustment rather than joint adjustment) using the *survey* package in R. Post-stratification adjustments were not done for the outpatient clinical cohort or the nursing home cohort due to unavailability of census data on these target populations. Individuals were formed into cells based on known demographic information. In each adjustment cell, the weighted estimates of the demographic variables were calculated using the existing survey weights and set to match the known population total using the most recent 5-year (2015-2019) county-level estimates from the American Community Survey (ACS) (https://data.census.gov/table/?d=ACS%205-Year%20Estimates%20Detailed%20Tables). If a catchment area cut across county lines, the estimates of all the counties in the catchment area were used for each post-stratification factor: age group, sex, race, and ethnicity. For by-age analyses, the adjustment cells were only matched to the known margins for that particular age category and did not use age as a further post-stratification factor. When using race as a post-

stratification factor, we used the three categories of Black, White and Other defined previously by collapsing race categories from the ACS.

If the selection probabilities or estimated response propensities were close to zero or one, the resulting design-based estimators were likely to be unstable. For this reason, trimming was done by censoring the weights at -/+ 4*IQR for a given analysis.

Additional Methods notes: Catchment areas: Two sites (New Orleans and Miami) recruited in zip codes beyond the contiguous zip codes surrounding the zip code of the clinical research site because the immediate surroundings had few residential areas.

Removal of records: A total of 456 participant records from the Tulane University site (Tulane) were removed from the analytic dataset after the Tulane Institutional Review Board (IRB) made a determination pertaining to certain data from the Tulane site. The IRB determined that data were not to be used for participants meeting both of the following criteria: participants recruited by staff who self-enrolled in the study and whose participation was not able to be subsequently verified.

The study did not track the number of people approached at nursing homes due to the complexity of recruitment from nursing homes during the earlier phase of the pandemic.

Ethics approvals: This study was conducted under the oversite of a central IRB, Advarra.

The following 10 sites submitted a waiver of oversight from their local IRB to Advarra as part of their submission. The sites, site PI and local IRB names are:

- CAIMED Center CRS / Elizabeth Barranco Santana: Ponce Medical School Foundation IRB
- University of Miami Infectious Disease Research Unit at Jackson Memorial Hospital CRS / Susanne Doblecki-Lewis: University of Miami IRB
- Penn Prevention CRS / Ian Frank: University of Pennsylvania IRB
- University of Pittsburgh CRS / Ken Ho: University of Pittsburgh IRB
- The Ponce de Leon Center CRS / Colleen Kelley: Emory IRB

- New Orleans Adolescent Trials Unit CRS/ Patricia Kissinger: Tulane University Biomedical IRB
- UIC Project WISH CRS / Stockton Mayer: UIC IRB
- Cincinnati CRS/ Margaret Powers-Fletcher: UC IRB
- Children’s Hospital Colorado CRS / Daniel Reirden: Colorado Multiple Institutional Review Board
- Columbia P&S CRS / Magdalena Sobieszczyk: CU CIRB

Five (5) sites did not submit a waiver and required additional approval from their local IRB. These sites, site PI and local IRB names are:

- Baylor College of Medicine CRS / Chris Amos: Baylor College of Medicine IRB
- Johns Hopkins University CRS / Jason Farley: Johns Hopkins University School of Medicine Institutional Review Board
- Harlem Prevention Center CRS / Yael Hirsch-Moverman: Columbia University Irving Medical Center IRB
- Bronx Prevention Research Center CRS / Jessica Justman: Columbia University Irving Medical Center IRB
- New Jersey Medical School Clinical Research Center CRS / Shobha Swaminathan: Rutgers University IRB

References

1. U.S. Food and Drug Administration. EUA Authorized Serology Test Performance 2022 [cited DATE]. https://www.fda.gov/medical-devices/covid-19-emergency-use-authorizations-medical-devices/eua-authorized-serology-test-performance

2. Peluso MJ, Takahashi S, Hakim J, Kelly JD, Torres L, Iyer NS, et al. SARS-CoV-2 antibody magnitude and detectability are driven by disease severity, timing, and assay. Sci Adv. 2021;7:eabh3409. PubMed https://doi.org/10.1126/sciadv.abh3409

3. Di Germanio C, Simmons G, Kelly K, Martinelli R, Darst O, Azimpouran M, et al. SARS-CoV-2 antibody persistence in COVID-19 convalescent plasma donors: Dependency on assay format and

applicability to serosurveillance. Transfusion. 2021;61:2677–87. PubMed https://doi.org/10.1111/trf.16555

4. Zangeneh SZ, Skalland T, Yuhas K, Emel L, Tapsoba JD, Reed D, et al. Adaptive Time Location Sampling for COMPASS, A SARS-COV-2 prevalence study in fifteen diverse communities in the United States. Epidemiology. 2023 Dec 12. Epub ahead of print. https://doi.org/10.1097/EDE.0000000000001705. **PMID: 38079239**.

Appendix Table 1. Enrollment by cohort by site, COMPASS 2021

Site	Cohort	N Approached[1]	N Enrolled	N Combined Analysis[2] Cohort
Atlanta	Community Venues	2540	1489	1318
	Outpatient Clinics	260	150	149
	Nursing Homes	.	.	.
Aurora, CO	Community Venues	2425	839	801
	Outpatient Clinics	.	.	.
	Nursing Homes	.	.	.
Baltimore	Community Venues	1148	877	812
	Outpatient Clinics	173	145	145
	Nursing Homes	.	.	.
Bronx	Community Venues	3659	2546	2338
	Outpatient Clinics	15	14	14
	Nursing Homes	.	.	.
Chicago	Community Venues	10426	1246	1187
	Outpatient Clinics	2756	335	335
	Nursing Homes	.	111	110
Cincinnati	Community Venues	7185	1188	1158
	Outpatient Clinics	245	35	35
	Nursing Homes	NA	12	12
Harlem	Community Venues	9704	2672	2553
	Outpatient Clinics	1502	503	502
	Nursing Homes	.	.	.
Houston	Community Venues	4399	1174	1146
	Outpatient Clinics	371	151	151
	Nursing Homes	.	32	32
Miami	Community Venues	2939	1480	1412
	Outpatient Clinics	.	.	.
	Nursing Homes	.	.	.
New Orleans	Community Venues	7831	1799	1765
	Outpatient Clinics	253	73	73
	Nursing Homes	.	201	201
NY Columbia	Community Venues	750	508	472
	Outpatient Clinics	342	265	265
	Nursing Homes	.	.	.
Newark	Community Venues	1673	861	820
	Outpatient Clinics	1085	262	261
	Nursing Homes	.	.	.
Philadelphia	Community Venues	6780	1564	1543
	Outpatient Clinics	1646	539	538
	Nursing Homes	.	.	.
Pittsburgh	Community Venues	2434	1117	1043
	Outpatient Clinics	420	139	139
	Nursing Homes	.	47	47
Ponce, Puerto Rico	Community Venues	4851	2924	2821
	Outpatient Clinics	1157	500	500
	Nursing Homes	.	403	403

Notes:
[1]Number of people approached as reported by each research site. Sites were not required to report number of people approached from nursing homes or senior living facilities.
[2]Number of people with complete data available for analysis of a combined Ab+ or PCR+ endpoint.

Appendix Table 2. Demographic characteristics of participants enrolled from community venues, by site, recruited from participating nursing homes and senior residential facilities, COMPASS 2021 (N = 22,284)

Characteristic	Atlanta % (n)	Aurora, CO % (n)	Baltimore % (n)	Bronx % (n)	Chicago % (n)	Cincinnati % (n)	Harlem % (n)	Houston % (n)	Miami % (n)	New Orleans % (n)	New York - Columbia % (n)	Newark % (n)	Philadel-phia % (n)	Pittsburgh % (n)	Ponce, Puerto Rico % (n)
Enrollments, N	1489	839	877	2546	1246	1188	2672	1174	1480	1799	508	861	1564	1117	2924
Age (years)															
<18	3% (47)	11% (92)	3% (22)	18% (460)	1% (15)	5% (59)	15% (411)	4% (49)	4% (54)	6% (108)	0% (0)	2% (18)	2% (29)	2% (22)	25% (727)
18 - 39	40% (591)	41% (342)	21% (180)	27% (677)	31% (385)	42% (503)	28% (761)	34% (403)	31% (460)	32% (571)	37% (189)	21% (184)	29% (458)	34% (377)	25% (732)
40 - 59	35% (525)	37% (312)	51% (444)	32% (802)	48% (602)	35% (410)	29% (762)	43% (507)	41% (603)	33% (590)	38% (191)	51% (435)	46% (724)	32% (353)	25% (736)
60+	22% (325)	11% (93)	26% (231)	24% (603)	20% (244)	18% (216)	28% (736)	18% (215)	24% (362)	29% (528)	25% (127)	26% (221)	23% (352)	33% (365)	25% (729)
Missing	0% (1)	0% (0)	0% (0)	0% (4)	0% (0)	0% (0)	0% (2)	0% (0)	0% (1)	0% (2)	0% (1)	0% (3)	0% (1)	0% (0)	0% (0)
Sex															
Female	45% (666)	51% (430)	48% (425)	51% (1296)	36% (443)	55% (653)	49% (1320)	61% (720)	45% (672)	48% (871)	59% (302)	47% (407)	40% (618)	52% (584)	59% (1715)
Male	54% (807)	49% (409)	52% (452)	49% (1246)	64% (803)	45% (533)	51% (1350)	39% (454)	54% (806)	51% (924)	40% (205)	52% (451)	60% (945)	47% (529)	41% (1205)
Missing	1% (16)	0% (0)	0% (0)	0% (4)	0% (0)	0% (2)	0% (2)	0% (0)	0% (2)	0% (4)	0% (1)	0% (3)	0% (1)	0% (4)	0% (4)
Race															
Black or African American	44% (649)	25% (213)	89% (781)	35% (900)	45% (562)	22% (258)	60% (1595)	37% (434)	27% (394)	64% (1152)	19% (95)	78% (672)	65% (1013)	22% (249)	9% (255)
White	40% (595)	39% (331)	6% (49)	11% (282)	19% (241)	67% (796)	7% (180)	55% (645)	64% (940)	28% (497)	28% (140)	9% (74)	26% (402)	64% (719)	80% (2340)
Other	13% (192)	34% (282)	4% (31)	47% (1195)	33% (406)	11% (125)	31% (834)	8% (91)	8% (122)	8% (137)	48% (244)	11% (92)	9% (142)	12% (130)	9% (261)
Prefer not to answer/Don't know	3% (41)	2% (13)	2% (16)	3% (83)	2% (30)	0% (4)	2% (46)	0% (3)	1% (19)	0% (6)	5% (27)	2% (20)	0% (6)	1% (15)	1% (41)
Missing	1% (12)	0% (0)	0% (0)	3% (86)	1% (7)	0% (5)	1% (17)	0% (1)	0% (5)	0% (7)	0% (2)	0% (3)	0% (1)	0% (4)	1% (27)
Ethnicity															
Hispanic or Latino	14% (207)	37% (308)	3% (30)	68% (1719)	38% (478)	5% (56)	33% (872)	38% (451)	67% (991)	7% (134)	56% (284)	16% (136)	9% (138)	8% (91)	98% (2880)
Not Hispanic or Latino	79% (1171)	62% (522)	90% (793)	31% (798)	60% (749)	94% (1119)	65% (1748)	61% (713)	32% (476)	91% (1645)	43% (216)	82% (706)	91% (1417)	87% (973)	1% (15)
Prefer not to answer/Don't know	2% (36)	1% (7)	6% (49)	1% (23)	1% (15)	0% (2)	1% (28)	0% (5)	0% (6)	1% (10)	1% (7)	1% (11)	0% (4)	3% (29)	0% (2)
Missing	5% (75)	0% (2)	1% (5)	0% (6)	0% (4)	1% (11)	1% (24)	0% (5)	0% (7)	1% (10)	0% (1)	1% (8)	0% (5)	2% (24)	1% (27)
Education															
No formal education	1% (15)	2% (13)	1% (11)	3% (87)	1% (8)	0% (2)	4% (110)	0% (5)	2% (23)	1% (22)	0% (2)	2% (20)	0% (5)	1% (6)	4% (117)
Pre-kindergarten	0% (2)	0% (1)	0% (1)	1% (34)	0% (0)	0% (2)	1% (40)	0% (3)	0% (2)	0% (8)	0% (0)	0% (1)	0% (1)	0% (0)	2% (52)
Kindergarten	1% (8)	1% (5)	0% (1)	4% (102)	0% (5)	0% (1)	4% (106)	0% (3)	1% (15)	2% (27)	0% (1)	0% (3)	0% (4)	0% (0)	2% (68)

Characteristic	Atlanta<br>% (n)	Aurora, CO<br>% (n)	Baltimore<br>% (n)	Bronx<br>% (n)	Chicago<br>% (n)	Cincinnati<br>% (n)	Harlem<br>% (n)	Houston<br>% (n)	Miami<br>% (n)	New Orleans<br>% (n)	New York - Columbia<br>% (n)	Newark<br>% (n)	Philadel-phia<br>% (n)	Pittsburgh<br>% (n)	Ponce, Puerto Rico<br>% (n)
Elementary school	1% (18)	6% (52)	1% (12)	10% (246)	2% (29)	1% (9)	5% (124)	4% (47)	4% (64)	2% (36)	2% (11)	1% (10)	1% (21)	0% (5)	9% (258)
Middle school	6% (83)	15% (122)	23% (202)	27% (677)	17% (215)	7% (89)	12% (324)	18% (216)	14% (207)	13% (236)	8% (40)	12% (106)	10% (162)	3% (31)	14% (414)
High school diploma/GED	25% (375)	28% (237)	53% (462)	31% (792)	42% (528)	28% (336)	38% (1008)	33% (393)	47% (689)	33% (599)	22% (114)	52% (448)	61% (952)	22% (244)	24% (705)
Some college/university	16% (236)	19% (156)	15% (135)	12% (314)	19% (236)	18% (216)	16% (419)	20% (229)	16% (241)	20% (359)	17% (88)	18% (152)	14% (217)	22% (242)	15% (426)
College/university degree	30% (445)	22% (183)	5% (43)	10% (243)	13% (168)	29% (340)	16% (422)	18% (210)	14% (206)	20% (358)	30% (151)	10% (90)	10% (158)	29% (324)	23% (663)
Post-graduate degree	20% (294)	8% (70)	1% (10)	2% (40)	5% (57)	16% (192)	4% (117)	6% (68)	2% (29)	8% (152)	20% (100)	3% (28)	3% (43)	23% (259)	7% (198)
Missing	1% (13)	0% (0)	0% (0)	0% (11)	0% (0)	0% (1)	0% (2)	0% (0)	0% (4)	0% (2)	0% (1)	0% (3)	0% (1)	1% (6)	1% (23)
Household income															
<$15,000	12% (173)	23% (196)	40% (351)	25% (645)	33% (417)	12% (141)	33% (883)	34% (394)	23% (347)	29% (517)	22% (111)	28% (238)	40% (625)	14% (160)	40% (1177)
$15,000 - $24,999	4% (66)	8% (69)	12% (108)	9% (222)	13% (158)	4% (47)	11% (285)	12% (139)	12% (185)	9% (153)	11% (56)	7% (59)	11% (165)	9% (97)	14% (409)
$25,000 - $34,999	5% (74)	6% (51)	5% (43)	5% (140)	8% (103)	4% (47)	8% (216)	10% (116)	5% (81)	6% (110)	7% (37)	6% (48)	5% (81)	7% (73)	7% (218)
$35,000 - $49,999	6% (87)	7% (60)	4% (36)	5% (128)	6% (71)	7% (86)	7% (199)	7% (85)	6% (83)	7% (123)	10% (49)	4% (36)	5% (73)	8% (88)	4% (123)
$50,000 – $74,999	8% (115)	8% (69)	2% (21)	3% (84)	5% (63)	9% (106)	6% (159)	6% (71)	4% (54)	6% (111)	15% (75)	4% (35)	5% (82)	11% (126)	3% (77)
$75,000 - $99,999	6% (88)	5% (44)	1% (8)	2% (39)	3% (34)	8% (98)	2% (56)	3% (37)	2% (24)	5% (85)	6% (32)	2% (21)	2% (37)	7% (82)	1% (42)
$100,000 - $149,999	8% (122)	8% (71)	1% (6)	1% (20)	3% (32)	9% (107)	2% (60)	4% (45)	1% (21)	4% (65)	10% (51)	1% (8)	2% (24)	9% (106)	1% (25)
$150,000 - $199,999	5% (71)	3% (28)	0% (3)	0% (4)	1% (9)	4% (53)	1% (30)	1% (16)	1% (10)	2% (32)	4% (18)	0% (2)	1% (14)	5% (53)	0% (2)
>$200,000	6% (93)	5% (43)	1% (5)	0% (1)	1% (17)	3% (31)	1% (27)	2% (28)	1% (12)	1% (21)	3% (14)	1% (7)	1% (10)	5% (55)	1% (20)
Don’t Know/Not sure	8% (124)	21% (178)	23% (203)	42% (1064)	24% (297)	32% (376)	24% (651)	12% (140)	29% (434)	25% (446)	8% (43)	28% (244)	24% (379)	15% (165)	22% (650)
Prefer not to answer	30% (442)	4% (30)	10% (91)	8% (193)	4% (45)	8% (94)	4% (104)	9% (103)	15% (224)	7% (134)	4% (21)	19% (160)	5% (73)	10% (107)	5% (140)
Missing	2% (34)	0% (0)	0% (2)	0% (6)	0% (0)	0% (2)	0% (2)	0% (0)	0% (5)	0% (2)	0% (1)	0% (3)	0% (1)	0% (5)	1% (41)
Employment (13 years/older)															
Employed - full time	44% (564)	39% (310)	14% (117)	15% (334)	22% (271)	42% (483)	19% (447)	32% (338)	30% (432)	28% (478)	40% (199)	22% (181)	18% (273)	42% (453)	26% (627)
Employed - part time	14% (176)	16% (130)	7% (61)	12% (262)	15% (183)	16% (184)	11% (248)	8% (85)	12% (171)	11% (183)	16% (80)	8% (65)	10% (152)	17% (181)	12% (273)
Homemaker/stay at home parent	2% (27)	2% (16)	0% (2)	2% (40)	1% (9)	1% (8)	2% (46)	4% (41)	3% (36)	1% (20)	1% (7)	1% (5)	1% (17)	1% (8)	13% (305)

Characteristic	Atlanta % (n)	Aurora, CO % (n)	Baltimore % (n)	Bronx % (n)	Chicago % (n)	Cincinnati % (n)	Harlem % (n)	Houston % (n)	Miami % (n)	New Orleans % (n)	New York - Columbia % (n)	Newark % (n)	Philadel-phia % (n)	Pittsburgh % (n)	Ponce, Puerto Rico % (n)
Not currently employed	21% (266)	29% (227)	34% (288)	43% (935)	39% (478)	25% (288)	44% (1031)	44% (467)	34% (479)	30% (510)	18% (93)	48% (399)	46% (718)	13% (137)	20% (467)
Retired	11% (141)	7% (55)	10% (86)	12% (273)	9% (108)	11% (120)	14% (332)	9% (98)	10% (143)	18% (305)	14% (72)	10% (84)	9% (146)	20% (213)	20% (466)
Disable	6% (77)	6% (45)	29% (248)	15% (338)	14% (170)	4% (43)	9% (202)	3% (37)	8% (119)	10% (163)	8% (41)	11% (94)	15% (232)	6% (61)	4% (87)
Other	2% (28)	1% (7)	5% (40)	1% (17)	1% (14)	1% (12)	2% (42)	1% (7)	3% (47)	2% (40)	2% (11)	1% (9)	1% (10)	3% (35)	6% (147)
Household size															
1	17% (254)	13% (110)	8% (68)	14% (360)	17% (207)	25% (297)	23% (603)	12% (146)	17% (252)	29% (521)	20% (104)	26% (225)	1% (14)	15% (162)	12% (341)
2	29% (427)	22% (184)	26% (228)	24% (602)	26% (328)	31% (369)	26% (702)	21% (252)	21% (314)	28% (497)	32% (163)	25% (212)	28% (437)	34% (380)	24% (712)
3-5	31% (457)	41% (343)	31% (271)	40% (1028)	36% (449)	35% (415)	38% (1011)	38% (449)	42% (615)	29% (513)	39% (199)	30% (256)	31% (491)	31% (343)	58% (1698)
>=6	3% (51)	15% (123)	6% (54)	13% (321)	8% (97)	4% (53)	7% (200)	10% (119)	8% (114)	5% (87)	3% (16)	9% (77)	9% (137)	4% (45)	3% (74)
Missing	20% (300)	9% (79)	29% (256)	9% (235)	13% (165)	5% (54)	6% (156)	18% (208)	12% (185)	10% (181)	5% (26)	11% (91)	31% (485)	17% (187)	3% (99)
Medical condition															
None	39% (583)	42% (355)	24% (207)	43% (1089)	36% (444)	53% (629)	43% (1146)	43% (510)	49% (726)	34% (618)	38% (195)	27% (235)	25% (396)	33% (367)	48% (1410)
At least one	30% (454)	57% (479)	76% (666)	57% (1447)	64% (796)	46% (549)	57% (1521)	56% (661)	50% (747)	65% (1171)	61% (309)	72% (622)	74% (1165)	66% (735)	51% (1484)
Missing	30% (452)	1% (5)	0% (4)	0% (10)	0% (6)	1% (10)	0% (5)	0% (3)	0% (7)	1% (10)	1% (4)	0% (4)	0% (3)	1% (15)	1% (30)
Medical conditions															
0	39% (583)	42% (355)	24% (207)	43% (1089)	36% (444)	53% (629)	43% (1146)	43% (510)	49% (726)	34% (618)	38% (195)	27% (235)	25% (396)	33% (367)	48% (1410)
1	20% (293)	29% (244)	25% (218)	23% (585)	25% (314)	26% (311)	24% (652)	26% (310)	26% (391)	27% (482)	28% (144)	26% (223)	26% (406)	28% (311)	26% (770)
2-3	9% (140)	22% (182)	37% (322)	25% (630)	30% (375)	17% (199)	25% (665)	25% (291)	19% (287)	32% (568)	25% (127)	32% (274)	34% (530)	29% (319)	21% (619)
>=4	1% (21)	6% (53)	14% (126)	9% (232)	9% (107)	3% (39)	8% (204)	5% (60)	5% (69)	7% (121)	7% (38)	15% (125)	15% (229)	9% (105)	3% (95)
Missing	30% (452)	1% (5)	0% (4)	0% (10)	0% (6)	1% (10)	0% (5)	0% (3)	0% (7)	1% (10)	1% (4)	0% (4)	0% (3)	1% (15)	1% (30)

Appendix Table 3. Demographic characteristics of participants enrolled from outpatient clinical venues, by site, COMPASS 2021 (N = 3,111)

Characteristic	Atlanta % (n)	Baltimore % (n)	Bronx % (n)	Chicago % (n)	Cincinnati % (n)	Harlem % (n)	Houston % (n)	New Orleans % (n)	New York - Columbia % (n)	Newark % (n)	Philadel-phia % (n)	Pittsburgh % (n)	Ponce, Puerto Rico % (n)
Enrollments, N	150	145	14	335	35	503	151	73	265	262	539	139	500
Age (years)													
18 - 39	31% (46)	32% (47)	7% (1)	29% (96)	14% (5)	21% (106)	39% (59)	21% (15)	26% (70)	38% (100)	30% (162)	31% (43)	36% (180)
40 - 59	43% (64)	47% (68)	64% (9)	39% (132)	57% (20)	60% (301)	48% (72)	59% (43)	47% (125)	45% (118)	49% (264)	46% (64)	43% (216)
60+	27% (40)	21% (30)	29% (4)	32% (107)	29% (10)	19% (96)	13% (20)	21% (15)	26% (70)	17% (44)	21% (113)	23% (32)	21% (104)
Sex													
Female	67% (100)	54% (79)	64% (9)	63% (211)	66% (23)	65% (329)	44% (66)	21% (15)	62% (163)	69% (180)	40% (216)	45% (63)	54% (268)
Male	33% (50)	46% (66)	36% (5)	37% (124)	34% (12)	35% (174)	56% (85)	79% (58)	38% (102)	31% (82)	60% (323)	55% (76)	46% (232)
Race													
Black or African American	72% (108)	83% (121)	14% (2)	57% (192)	54% (19)	70% (354)	51% (77)	67% (49)	26% (70)	54% (142)	66% (354)	37% (52)	13% (64)
White	24% (36)	12% (18)	7% (1)	19% (62)	43% (15)	3% (14)	46% (69)	32% (23)	10% (27)	24% (62)	24% (131)	51% (71)	83% (416)
Other	3% (5)	2% (3)	71% (10)	21% (69)	3% (1)	26% (130)	3% (5)	1% (1)	55% (146)	18% (48)	10% (52)	10% (14)	4% (20)
Prefer not to answer/Don't know	1% (1)	1% (2)	7% (1)	2% (7)	0% (0)	1% (5)	0% (0)	0% (0)	8% (22)	3% (9)	0% (2)	1% (2)	0% (0)
Missing	0% (0)	1% (1)	0% (0)	1% (5)	0% (0)	0% (0)	0% (0)	0% (0)	0% (0)	0% (1)	0% (0)	0% (0)	0% (0)
Ethnicity													
Hispanic or Latino	9% (14)	1% (2)	57% (8)	29% (96)	0% (0)	25% (128)	28% (43)	4% (3)	65% (171)	26% (68)	9% (51)	9% (12)	99% (493)
Not Hispanic or Latino	86% (129)	92% (133)	36% (5)	71% (238)	97% (34)	73% (367)	71% (107)	95% (69)	35% (93)	73% (192)	90% (486)	88% (122)	0% (1)
Prefer not to answer/Don't know	1% (2)	5% (7)	7% (1)	0% (0)	0% (0)	1% (3)	0% (0)	0% (0)	0% (1)	1% (2)	0% (1)	4% (5)	0% (1)
Missing	3% (5)	2% (3)	0% (0)	0% (1)	3% (1)	1% (5)	1% (1)	1% (1)	0% (0)	0% (0)	0% (1)	0% (0)	1% (5)
Education													
No formal education	1% (2)	0% (0)	0% (0)	1% (2)	0% (0)	1% (4)	0% (0)	0% (0)	0% (0)	1% (2)	0% (2)	0% (0)	1% (7)
Kindergarten	0% (0)	0% (0)	0% (0)	0% (0)	0% (0)	0% (1)	0% (0)	1% (1)	0% (0)	0% (0)	0% (0)	0% (0)	1% (3)
Elementary school	1% (2)	0% (0)	7% (1)	1% (2)	0% (0)	2% (12)	3% (4)	0% (0)	3% (9)	0% (0)	0% (2)	0% (0)	5% (23)
Middle school	1% (2)	13% (19)	14% (2)	9% (31)	9% (3)	12% (60)	12% (18)	21% (15)	11% (28)	2% (6)	11% (59)	0% (0)	9% (43)
High school diploma/GED	21% (32)	52% (76)	36% (5)	29% (96)	40% (14)	41% (206)	35% (53)	38% (28)	28% (75)	32% (83)	56% (301)	22% (31)	26% (132)
Some college/university	18% (27)	22% (32)	29% (4)	32% (108)	37% (13)	18% (93)	28% (43)	23% (17)	22% (58)	27% (72)	16% (86)	29% (40)	22% (108)
College/university degree	35% (52)	10% (15)	7% (1)	23% (77)	11% (4)	19% (96)	20% (30)	15% (11)	23% (61)	28% (74)	11% (61)	32% (44)	28% (142)

Characteristic	Atlanta % (n)	Baltimore % (n)	Bronx % (n)	Chicago % (n)	Cincinnati % (n)	Harlem % (n)	Houston % (n)	New Orleans % (n)	New York - Columbia % (n)	Newark % (n)	Philadel-phia % (n)	Pittsburgh % (n)	Ponce, Puerto Rico % (n)
Post-graduate degree	22% (33)	2% (3)	7% (1)	6% (19)	3% (1)	6% (31)	2% (3)	1% (1)	13% (34)	10% (25)	5% (28)	17% (24)	8% (42)
Household income													
<$15,000	3% (4)	23% (33)	57% (8)	23% (76)	17% (6)	21% (107)	34% (51)	34% (25)	20% (53)	10% (26)	29% (156)	14% (19)	50% (249)
$15,000 - $24,999	5% (7)	8% (11)	14% (2)	13% (43)	9% (3)	10% (51)	5% (8)	7% (5)	9% (24)	4% (11)	9% (50)	16% (22)	13% (64)
$25,000 - $34,999	3% (4)	9% (13)	0% (0)	9% (30)	14% (5)	6% (30)	5% (7)	5% (4)	8% (22)	9% (24)	6% (32)	9% (13)	8% (41)
$35,000 - $49,999	7% (10)	10% (15)	0% (0)	10% (35)	17% (6)	8% (40)	18% (27)	5% (4)	7% (19)	15% (39)	7% (38)	10% (14)	6% (30)
$50,000 – $74,999	14% (21)	6% (8)	0% (0)	15% (49)	6% (2)	9% (43)	6% (9)	4% (3)	15% (40)	12% (31)	4% (23)	6% (9)	3% (17)
$75,000 - $99,999	9% (14)	4% (6)	0% (0)	4% (15)	3% (1)	3% (16)	2% (3)	3% (2)	7% (18)	7% (19)	3% (18)	9% (13)	1% (4)
$100,000 - $149,999	7% (11)	0% (0)	0% (0)	4% (12)	3% (1)	2% (10)	1% (2)	0% (0)	8% (21)	9% (23)	2% (13)	7% (10)	1% (3)
$150,000 - $199,999	5% (8)	2% (3)	0% (0)	1% (4)	0% (0)	1% (4)	1% (2)	0% (0)	5% (13)	4% (11)	1% (8)	5% (7)	0% (1)
>$200,000	4% (6)	0% (0)	0% (0)	1% (3)	0% (0)	1% (3)	1% (2)	1% (1)	1% (3)	2% (4)	1% (3)	4% (5)	0% (1)
Don’t Know/Not sure	8% (12)	19% (27)	21% (3)	17% (56)	31% (11)	35% (174)	15% (23)	30% (22)	12% (33)	13% (33)	31% (169)	14% (20)	17% (83)
Prefer not to answer	35% (53)	20% (29)	7% (1)	4% (12)	0% (0)	5% (25)	11% (17)	10% (7)	7% (19)	16% (41)	5% (29)	5% (7)	1% (7)
Employment (13 years/older)													
Employed - full time	62% (79)	35% (51)	7% (1)	30% (100)	31% (11)	28% (142)	42% (62)	22% (16)	47% (122)	64% (165)	21% (114)	55% (75)	41% (203)
Employed - part time	5% (6)	6% (8)	0% (0)	11% (36)	6% (2)	9% (45)	7% (10)	1% (1)	8% (21)	6% (15)	11% (58)	9% (13)	8% (40)
Homemaker/stay at home parent	3% (4)	0% (0)	0% (0)	2% (7)	3% (1)	2% (10)	1% (2)	0% (0)	0% (0)	0% (0)	2% (12)	0% (0)	11% (54)
Not currently employed	9% (12)	31% (45)	14% (2)	28% (93)	20% (7)	43% (215)	40% (58)	58% (42)	24% (63)	21% (55)	42% (225)	12% (17)	10% (49)
Retired	16% (21)	6% (9)	7% (1)	13% (42)	14% (5)	8% (42)	4% (6)	8% (6)	10% (25)	2% (5)	8% (45)	12% (16)	9% (43)
Disable	4% (5)	19% (27)	71% (10)	16% (52)	23% (8)	9% (43)	4% (6)	11% (8)	10% (27)	4% (11)	16% (84)	11% (15)	14% (70)
Other	1% (1)	3% (5)	0% (0)	1% (4)	3% (1)	1% (6)	1% (2)	0% (0)	1% (3)	2% (6)	0% (1)	1% (1)	8% (39)
Household size													
1	19% (28)	9% (13)	36% (5)	19% (63)	26% (9)	17% (88)	17% (26)	32% (23)	24% (64)	14% (36)	1% (6)	9% (12)	8% (42)
2	23% (35)	21% (30)	14% (2)	27% (91)	29% (10)	28% (140)	17% (26)	15% (11)	26% (68)	27% (72)	26% (142)	29% (41)	27% (137)
3-5	40% (60)	40% (58)	29% (4)	36% (121)	29% (10)	36% (181)	36% (55)	30% (22)	40% (105)	45% (118)	40% (217)	29% (40)	39% (196)
>=6	5% (8)	12% (18)	0% (0)	7% (23)	17% (6)	7% (37)	5% (8)	8% (6)	5% (14)	8% (22)	5% (27)	1% (2)	21% (106)
Missing	13% (19)	18% (26)	21% (3)	11% (37)	0% (0)	11% (57)	24% (36)	15% (11)	5% (14)	5% (14)	27% (147)	32% (44)	4% (19)
Medical conditions													
None	25% (38)	19% (27)	0% (0)	16% (52)	29% (10)	35% (177)	50% (76)	16% (12)	24% (63)	37% (98)	23% (124)	15% (21)	33% (167)
At least one	36% (54)	81% (118)	100% (14)	84% (283)	71% (25)	65% (326)	50% (75)	84% (61)	75% (198)	62% (162)	77% (414)	83% (116)	67% (333)

Characteristic	Atlanta % (n)	Baltimore % (n)	Bronx % (n)	Chicago % (n)	Cincinnati % (n)	Harlem % (n)	Houston % (n)	New Orleans % (n)	New York - Columbia % (n)	Newark % (n)	Philadel-phia % (n)	Pittsburgh % (n)	Ponce, Puerto Rico % (n)
Missing	39% (58)	0% (0)	0% (0)	0% (0)	0% (0)	0% (0)	0% (0)	0% (0)	2% (4)	1% (2)	0% (1)	1% (2)	0% (0)
Medical conditions													
0	25% (38)	19% (27)	0% (0)	16% (52)	29% (10)	35% (177)	50% (76)	16% (12)	24% (63)	37% (98)	23% (124)	15% (21)	33% (167)
1	21% (32)	34% (50)	0% (0)	23% (77)	23% (8)	30% (152)	23% (35)	23% (17)	28% (73)	26% (68)	26% (140)	31% (43)	33% (167)
2-3	12% (18)	34% (50)	43% (6)	41% (136)	29% (10)	27% (135)	18% (27)	38% (28)	31% (83)	29% (76)	37% (199)	32% (44)	28% (141)
>=4	3% (4)	12% (18)	57% (8)	21% (70)	20% (7)	8% (39)	9% (13)	22% (16)	16% (42)	7% (18)	14% (75)	21% (29)	5% (25)
Missing	39% (58)	0% (0)	0% (0)	0% (0)	0% (0)	0% (0)	0% (0)	0% (0)	2% (4)	1% (2)	0% (1)	1% (2)	0% (0)

Appendix Table 4. Demographic characteristics of participants enrolled from nursing homes, by site, COMPASS 2021 (N = 806)

Characteristic	Chicago	Cincinnati	Houston	New Orleans	Pittsburgh	Ponce, Puerto Rico
	% (n)	% (n)	% (n)	% (n)	% (n)	% (n)
Enrollments, N	111	12	32	201	47	403
Age (years)						
18 - 39	9% (10)	8% (1)	0% (0)	0% (0)	2% (1)	0% (0)
40 - 59	30% (33)	0% (0)	0% (0)	10% (20)	2% (1)	2% (8)
60+	61% (68)	92% (11)	100% (32)	90% (181)	96% (45)	98% (395)
Sex						
Female	32% (35)	58% (7)	66% (21)	46% (93)	66% (31)	66% (266)
Male	68% (76)	42% (5)	34% (11)	54% (108)	34% (16)	34% (136)
Missing	0% (0)	0% (0)	0% (0)	0% (0)	0% (0)	0% (1)
Race						
Black or African American	51% (57)	0% (0)	0% (0)	89% (179)	0% (0)	7% (30)
White	35% (39)	92% (11)	100% (32)	3% (6)	98% (46)	90% (362)
Other	13% (14)	8% (1)	0% (0)	6% (13)	0% (0)	2% (10)
Prefer not to answer/Don't know	1% (1)	0% (0)	0% (0)	1% (3)	0% (0)	0% (0)
Missing	0% (0)	0% (0)	0% (0)	0% (0)	2% (1)	0% (1)
Ethnicity						
Hispanic or Latino	16% (18)	0% (0)	3% (1)	3% (6)	0% (0)	99% (399)
Not Hispanic or Latino	81% (90)	92% (11)	97% (31)	96% (192)	96% (45)	0% (0)
Prefer not to answer/Don't know	2% (2)	0% (0)	0% (0)	1% (2)	2% (1)	0% (1)
Missing	1% (1)	8% (1)	0% (0)	0% (1)	2% (1)	1% (3)
Education						
No formal education	0% (0)	0% (0)	0% (0)	0% (0)	0% (0)	4% (17)
Kindergarten	1% (1)	0% (0)	0% (0)	0% (0)	0% (0)	1% (3)
Elementary school	2% (2)	0% (0)	0% (0)	3% (6)	0% (0)	12% (49)
Middle school	12% (13)	0% (0)	0% (0)	23% (47)	0% (0)	17% (70)
High school diploma/GED	42% (47)	42% (5)	6% (2)	47% (94)	28% (13)	40% (161)
Some college/university	25% (28)	17% (2)	31% (10)	18% (37)	19% (9)	12% (48)
College/university degree	8% (9)	33% (4)	41% (13)	6% (12)	26% (12)	11% (43)
Post-graduate degree	6% (7)	8% (1)	22% (7)	2% (5)	26% (12)	3% (12)
Missing	4% (4)	0% (0)	0% (0)	0% (0)	2% (1)	0% (0)
Household income						
<$15,000	41% (46)	0% (0)	0% (0)	47% (95)	4% (2)	88% (354)
$15,000 - $24,999	10% (11)	8% (1)	6% (2)	14% (28)	2% (1)	4% (15)
$25,000 - $34,999	1% (1)	8% (1)	3% (1)	1% (2)	9% (4)	0% (1)
$35,000 - $49,999	2% (2)	8% (1)	9% (3)	0% (1)	4% (2)	0% (0)
$50,000 – $74,999	0% (0)	0% (0)	22% (7)	1% (2)	15% (7)	0% (0)
$75,000 - $99,999	0% (0)	0% (0)	16% (5)	0% (1)	11% (5)	0% (0)
$100,000 - $149,999	0% (0)	0% (0)	9% (3)	0% (0)	6% (3)	0% (0)
$150,000 - $199,999	1% (1)	0% (0)	9% (3)	0% (0)	2% (1)	0% (0)
>$200,000	1% (1)	0% (0)	6% (2)	0% (0)	0% (0)	0% (0)
Don't Know/Not sure	42% (47)	75% (9)	3% (1)	31% (62)	34% (16)	8% (33)
Prefer not to answer	2% (2)	0% (0)	16% (5)	5% (10)	11% (5)	0% (0)
Missing	0% (0)	0% (0)	0% (0)	0% (0)	2% (1)	0% (0)
Employment (13 years/older)						
Employed - full time	1% (1)	0% (0)	0% (0)	4% (7)	4% (2)	0% (1)
Employed - part time	0% (0)	8% (1)	0% (0)	2% (3)	0% (0)	1% (2)
Homemaker/stay at home parent	0% (0)	0% (0)	0% (0)	1% (1)	0% (0)	19% (73)
Not currently employed	35% (37)	0% (0)	13% (4)	6% (11)	2% (1)	2% (9)
Retired	32% (34)	92% (11)	87% (26)	56% (111)	93% (43)	65% (254)
Disable	33% (35)	0% (0)	0% (0)	32% (64)	0% (0)	8% (31)
Other	0% (0)	0% (0)	0% (0)	0% (0)	0% (0)	5% (18)
Household size						
1	15% (17)	67% (8)	62% (20)	77% (154)	19% (9)	67% (272)
2	50% (55)	25% (3)	19% (6)	8% (17)	45% (21)	7% (30)
3-5	21% (23)	8% (1)	0% (0)	0% (0)	0% (0)	1% (5)
>=6	0% (0)	0% (0)	0% (0)	0% (0)	0% (0)	19% (76)
Missing	14% (16)	0% (0)	19% (6)	15% (30)	36% (17)	5% (20)
Medical conditions						
None	5% (6)	25% (3)	16% (5)	14% (29)	17% (8)	14% (55)
At least one	88% (98)	58% (7)	84% (27)	86% (172)	81% (38)	81% (325)
Missing	6% (7)	17% (2)	0% (0)	0% (0)	2% (1)	6% (23)
Medical conditions						
0	5% (6)	25% (3)	16% (5)	14% (29)	17% (8)	14% (55)
1	5% (5)	17% (2)	31% (10)	28% (57)	28% (13)	23% (93)
2-3	31% (34)	42% (5)	31% (10)	37% (74)	45% (21)	46% (187)
>=4	53% (59)	0% (0)	22% (7)	20% (41)	9% (4)	11% (45)

Characteristic	Chicago % (n)	Cincinnati % (n)	Houston % (n)	New Orleans % (n)	Pittsburgh % (n)	Ponce, Puerto Rico % (n)
Missing	6% (7)	17% (2)	0% (0)	0% (0)	2% (1)	6% (23)

Appendix Table 5. Seroprevalence (Ab+) and percent of nasal samples SARS-CoV-2 PCR positive by cohort, across sites, COMPASS 2021

Cohort	PCR+ Median [IQR]	Ab+ Median [IQR]
Community	0.8% [0.2%, 1.5%]	12.4% [9.1%, 13.9%]
Clinical	0.4% [0%, 0.9%]	11.3% [7.7%, 15.5%]
Nursing Homes	0% [0%, 0%]	3.3% [2.4%, 7.7%]

Notes: the prevalence estimates for the community cohort were based on post-stratified weights while the estimates for the clinical cohort used design base weights and those for the nursing home cohort were unweighted.

Appendix Table 6: Combined active or prior infection prevalence and vaccine willingness (median, IQR) among the community cohort (N = 22,284)

Group	Combined Prevalence Median [IQR]	Vaccine Willingness Median [IQR]
Overall	12.9% [9.2%, 13.9%]	77.5% [72.1%, 82.2%]
Age (years)		
<18	10.8% [9.0%, 11.8%]	61.0% [52.3%, 72.3%]
18-39	9.9% [8.0%, 11.8%]	73.4% [66.7%, 82.9%]
40-59	12.2% [10.2%, 13.5%]	75.7% [74.0%, 81.0%]
60+	13.0% [7.7%, 14.7%]	88.8% [83.5%, 92.5%]
Sex		
Male	12.3% [9.9%, 13.2%]	76.3% [71.9%, 79.8%]
Female	12.0% [8.2%, 14.3%]	76.3% [71.5%, 83.1%]
Race		
Black	13.2% [11.7%, 15.2%]	71.4% [64.8%, 76.3%]
White	9.3% [6.1%, 10.9%]	84.2% [73.6%, 87.0%]
Other	13.4% [9.7%, 14.9%]	78.1% [68.8%, 82.5%]
Ethnicity		
Hispanic/Latino	15.4% [11.5%, 16.4%]	76.4% [70.3%, 80.5%]
Not Hispanic/Latino	10.6% [8.5%, 13.2%]	78.1% [71.7%, 85.0%]

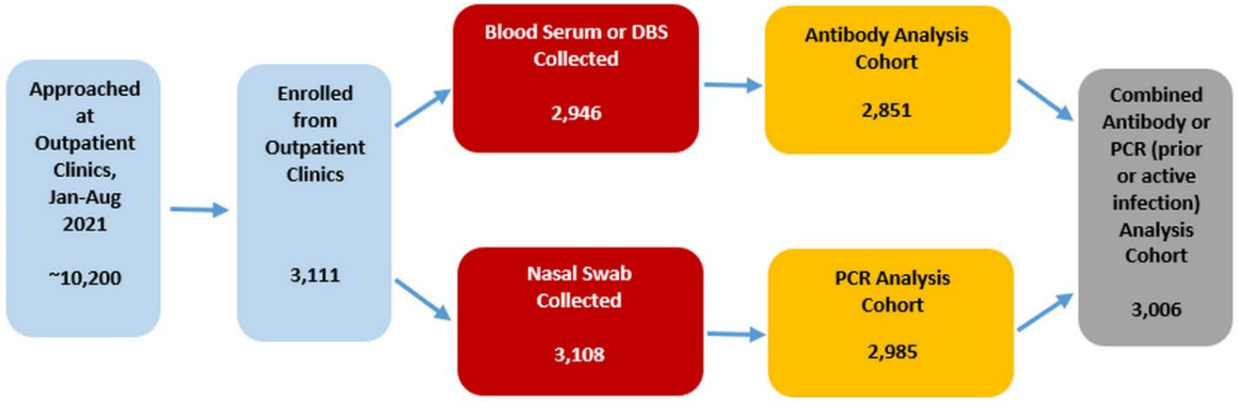

Appendix Figure 1. Participant enrollment from outpatient clinics, COMPASS 2021. DBS: dried blood sample.

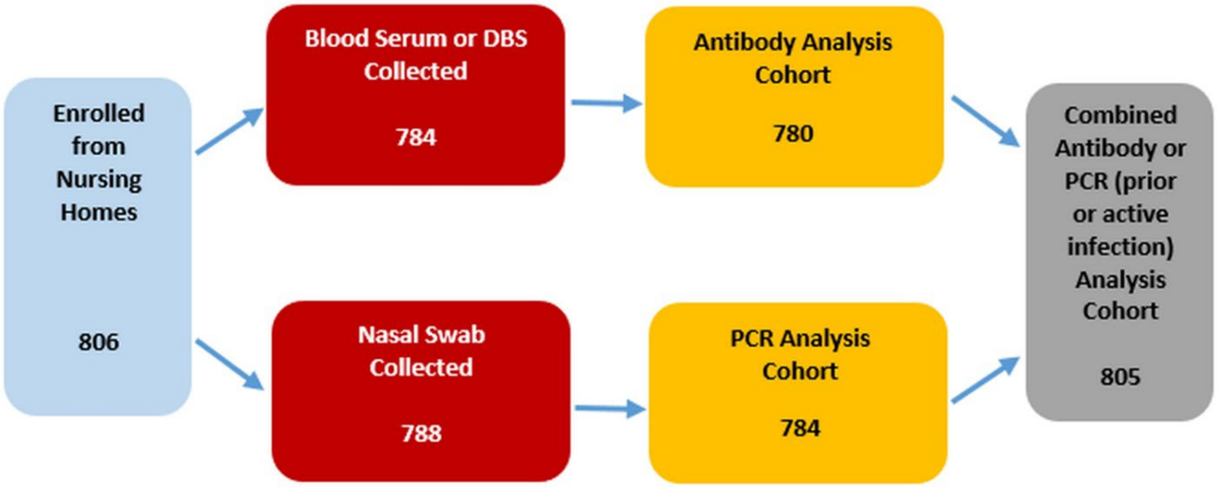

Appendix Figure 2. Participant enrollment from nursing homes, COMPASS 2021. Note: Sites were not required to report number of people approached from nursing homes or senior living facilities. DBS, dried blood spot.

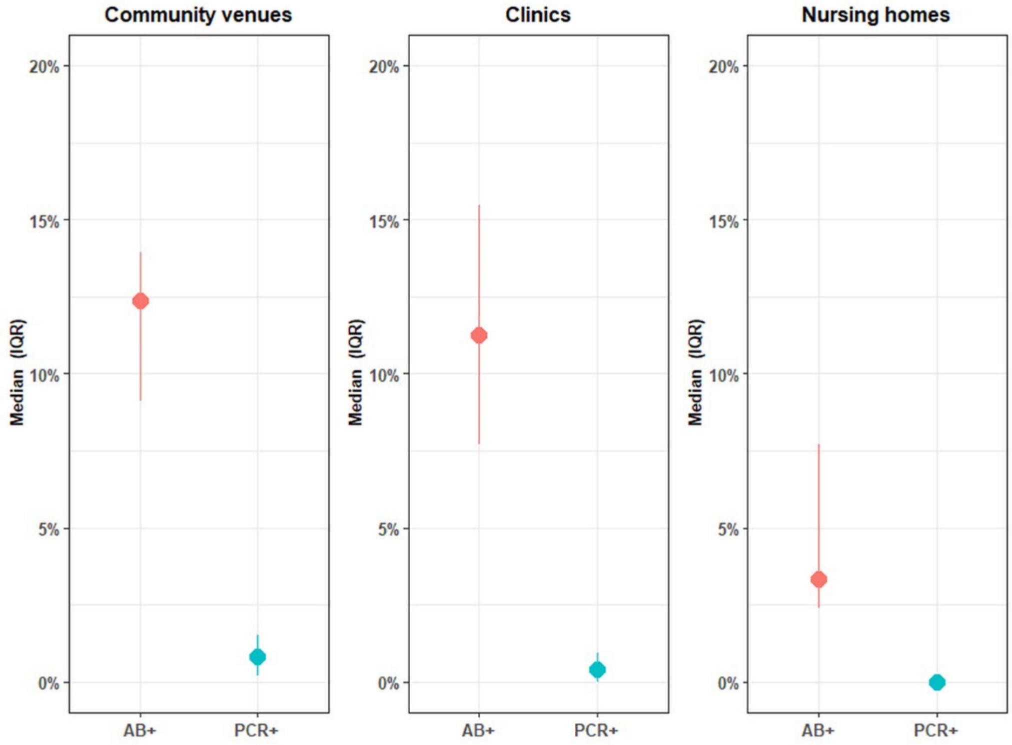

Appendix Figure 3. Seroprevalence and percent of nasal samples SARS-CoV-2 PCR positive by cohort, across sites, COMPASS 2021.

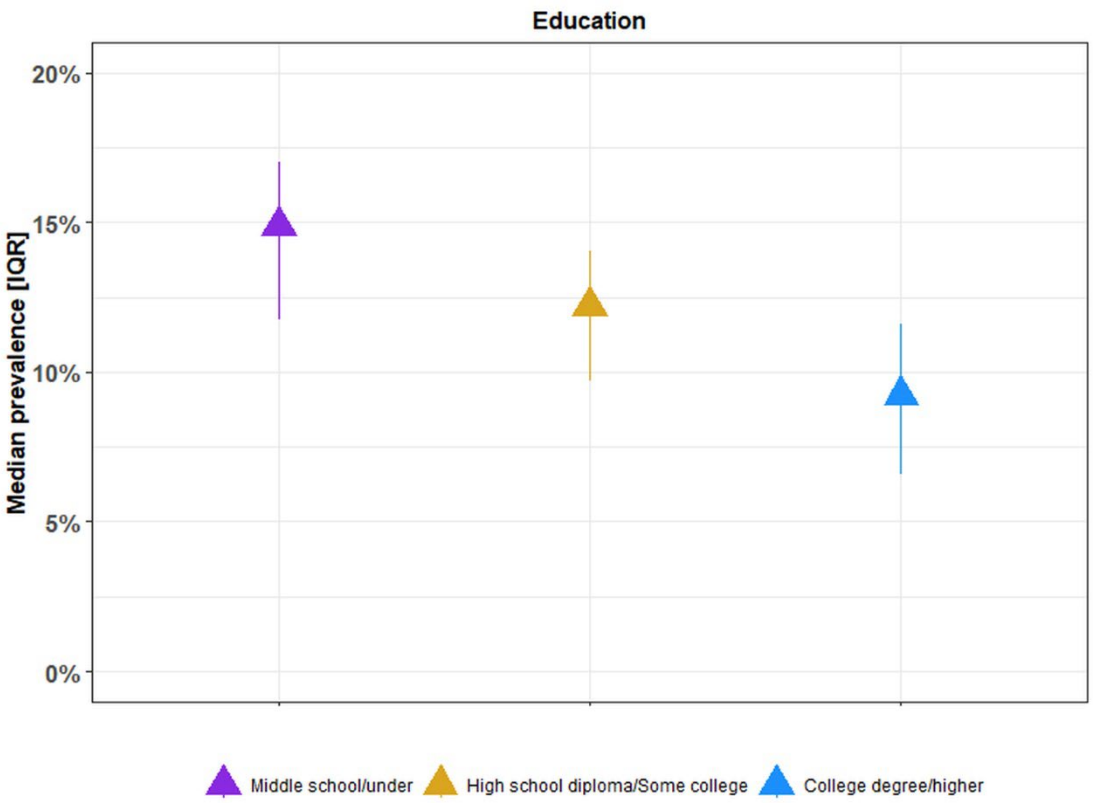

Appendix Figure 4. Prevalence of active or prior infection by education among those age 18 years and older in community cohort across all sites, COMPASS 2021.
